# Fathers’ caregiving time before and after the COVID-19 pandemic

**DOI:** 10.1371/journal.pone.0343636

**Published:** 2026-03-16

**Authors:** Lee T. Gettler, Sarah Hoegler Dennis, Stacy Rosenbaum, Sonny Agustin Bechayda, Christopher W. Kuzawa

**Affiliations:** 1 Department of Anthropology, University of Notre Dame, Notre Dame, Indiana, United States of America; 2 Eck Institute for Global Health, University of Notre Dame, Notre Dame, Indiana, United States of America; 3 Department of Psychology, University of Notre Dame, Notre Dame, Indiana, United States of America; 4 Department of Anthropology, University of Michigan, Ann Arbor, Michigan, United States of America; 5 USC Office of Population Studies Foundation, and Department of Anthropology, Sociology, and History, University of San Carlos, Metro Cebu, Philippines; 6 Department of Anthropology, Northwestern University, Evanston, Illinois, United States of America; 7 Institute for Policy Research, Northwestern University, Evanston, Illinois, United States of America; Curtin University Bentley Campus: Curtin University, AUSTRALIA

## Abstract

The COVID-19 pandemic contributed to greater day-to-day time spent together for many families. Some research in Euro-American settings indicated that fathers spent more time caring for their children during the pandemic than previously, with the potential for lasting effects on fathers’ caregiving. However, there are few longitudinal studies with post-pandemic paternal care data and analyses from non-Euro-American contexts. We drew on a large sample of men (N = 649; Obs. = 1286) enrolled in a longitudinal study in the Philippines with waves of data collection prior to the pandemic in 2009 (wave 1) and 2014 (wave 2) and a third wave post-pandemic in 2022−23 (wave 3). Our main analyses focus on within-individual change in paternal caregiving for men residing with young children (N = 307) from the pre-pandemic (wave 2) to post-pandemic period (wave 3). Fathers showed meaningful within-individual declines in routine (intensive) caregiving time from the pre-pandemic (wave 2) to the post-pandemic (wave 3). However, they reported comparable engagement in overall, recreational, and educational care post-pandemic relative to their pre-pandemic levels at wave 2. Men’s shifts in pre-to-post-pandemic employment status predicted their changes in caregiving time, particularly for educational care. The post-pandemic patterns we observed for educational care are potentially consistent with shifting dynamics for fathers’ caregiving in this domain following the pandemic and complement results elsewhere connecting fathers’ employment characteristics to their caregiving during and after the pandemic.

## Introduction

The COVID-19 global pandemic affected family dynamics in societies around the world through a variety of social, economic, and health pathways. Economic shutdowns and the transition to work-from-home arrangements for certain professions led to a period of more intensive day-to-day time spent together for many families. In Euro-American settings, there was significant media coverage, and some early empirical evidence, suggesting that fathers were spending more time caring for their children during the lockdown period than prior to the pandemic, though mothers still typically bore greater responsibility for care and household tasks [1,2]. Viewed through the lens of the socio-ecological model [3,4], the pandemic could be framed as changing multiple levels of the ecological system. Given the scope of those alterations, there is potential for lasting, post-pandemic effects on fathers’ caregiving roles in family systems in some settings and the possibility of increasing gender equity in childcare responsibilities in families with different-gender parents.

However, the ramifications of the pandemic for family systems and their post-pandemic persistence are likely to be variable across- and within-societal contexts, differing by political economic, cultural, and family characteristics [1,5]. For example, pandemic-driven governmental policies and labor dynamics might only shape long-term shifts in fathering in societal contexts with prolonged lockdowns or labor markets with ample work-from-home opportunities for fathers. This would be particularly applicable if the primary reason fathers do not provide more care is because they are time constrained [6,7]. Even within the same society, discrepancies in the flexibility to work from home by socioeconomic status may have similarly influenced whether fathers and their children experienced sustained increases in time together across the pandemic [8,9]. Mothers’ employment dynamics, availability of alloparental caregivers, and parents’ gender ideologies and stereotypes could also have affected the pandemic’s influence on household divisions of labor in families with different-gender parents [8,10–12]. The amount of time that fathers spend on caregiving is not an indicator of the quality of the care they provide [13–15]. However, positive child development outcomes linked to specific forms of parental involvement are conceptualized as an interaction between both quality and the quantity of that care [13,15,16], and fathers’ time spent on childcare and household tasks is relevant to well-being in families with different-gender parents [17,18].

Existing research on Euro-American family dynamics during the pandemic provides evidence of such variation in family divisions of childcare labor, particularly related to employment. In research using cross-sectional time diary data from nationally-representative U.S. samples, fathers and mothers working from home during the pandemic reported spending more time with their children and directly engaging in childcare compared to work-from-home parents prior to the pandemic. Parents working outside the home before and during the pandemic did not have equivalent differences for their time spent with children and in caregiving [8]. In a study using the same U.S. data, researchers found that the overall gender gap in childcare diminished modestly during the pandemic. However, the gap grew for intensive caregiving demands that parents typically find more demanding or stressful [19], which can include educational activities (e.g., homework) or routine, inflexible physical care tasks for young children [20]. During the pandemic period, parents were required to dedicate greater time to children’s educational activities due to online learning. These increased demands and the time constraints imposed on parents to manage their children’s online learning were linked to poorer parental mental well-being [21,22], highlighting that increased time availability for childcare and household tasks that can accompany work-from-home flexibility can also enhance demands on parents, depending on context and family system dynamics [21–23]. Moreover, the responsibility for educational caregiving tasks during the pandemic often fell disproportionately on mothers, though studies differ in whether the gender gap grew for this domain during this period [8,19].

Two longitudinal studies following families across the first year of the pandemic in the U.S. and Germany, respectively, found parallel results: fathers reported being more involved with childcare during the pandemic, particularly in the immediate lockdown period, compared to their retrospective reports of pre-pandemic caregiving [24,25]. In the U.S., fathers working exclusively from home reported the most childcare involvement, and fathers’ care also varied based on whether mothers were in the paid labor force or worked from home [25]. In the German findings, both mothers’ and fathers’ involvement varied similarly by family employment dynamics (i.e., work-from-home status; partner labor force participation) [24]. While notable, such results do not speak to whether these behavioral changes persisted post-pandemic. In one of the only longitudinal analyses that included more recent data (i.e., beyond 2022), fathers’ increased childcare involvement only persisted following the pandemic among a relatively small percentage of U.S. families who shifted from a pre-pandemic, ‘mother-as-primary-caregiver’ arrangement to a more ‘equally-shared’ or ‘father-as-primary-caregiver’ arrangement during the pandemic [26]. In total, a number of gaps remain in this research domain, including few longitudinal studies with post-pandemic paternal care data that can inform on durable changes in parenting roles and sparse information on these dynamics from non-Euro-American contexts, where family dynamics differ.

In the present study, we draw on data from a long-running birth cohort study in the Philippines to help address questions regarding paternal care before and after the pandemic period. The Philippines is a lower-middle-income (LMIC) nation that is home to over 110 million people. Due to its long history of experiencing Spanish colonialism and the associated influence of the Catholic church, the country is overwhelmingly Catholic [27]. Historically, the influence of Catholicism has contributed to dynamics related to family life, such as divorce being illegal, contraception being limited (i.e., in availability and adoption), and family size and fertility being higher than in many other LMICs in the region [27–30]. However, over the past few decades the fertility rate per woman has declined from 4.1 children per woman in 1993 to 1.9 children per woman in 2022, with fertility rates remaining higher among the poorest households [30]. Traditionally, Catholic norms have likewise helped shape valued local roles for fathers and Filipino cultural emphases on two-parent households [28].

From a macro-system socio-ecological perspective, this is a pertinent context for these pandemic-focused questions because the Philippines experienced one of the world’s longest shutdown periods, lasting more than two years, including delayed resumption of in-person schooling, which provided an extended window of time for pandemic-related effects on parenting roles. The pandemic also coincided with and accelerated growth in remote, work-from-home opportunities for Filipino workers [31], although access to such jobs remains unequal by socioeconomic class and region, as only a slight majority of Filipino households had internet access as of 2020, including in the region where the present study took place [32]. These internet access constraints along with similar disparities in online-learning device availability (i.e., computers; tablets) also shaped the educational landscape for Filipino families during the pandemic period [33]. The Department of Education in the Philippines offered multiple options for student learning during the pandemic, with families most commonly adopting the modular approach that required the use of printed or (less commonly) digital materials for students to engage in “self-learning modules” with parents serving as the main instructors at home. To assess progress, teachers then periodically retrieved the schoolwork that students produced at home, under the supervision of their parents or other caregivers [33]. Thus, parents played primary instructor roles for at-home learning during the pandemic through a mixture of modalities.

In prior generations, Filipino fathers’ roles commonly centered on providing resources and being the symbolic head of the household, which includes instilling moral values in their children and ensuring they avoid behavioral problems [28,34], and they engaged in very little caregiving, on average [35,36]. In the past, mothers were the primary caregivers to children and older female children and other female kin often assisted with childcare, more so than fathers [28,36,37]. However, in the contemporary generation of Filipino families, there has been a shift towards greater involvement by fathers in childcare, on average. Nonetheless, there remains substantial variation in fathers’ caregiving roles across families [34,35,38]. This shift in gender dynamics around caregiving has coincided with increased educational achievement and labor opportunities outside the home for women, which has reshaped how families must navigate the demands of caring for young children [39,40]. Relevant to divisions of Filipino family labor during the pandemic, past work has shown that better-educated fathers and fathers engaged in less work outside the home, respectively, tend to be more involved with childcare [35,38,39].

Here, we used longitudinal data collected at multiple waves over almost fifteen years to investigate whether co-residential fathers’ caregiving time was different post-pandemic compared to pre-pandemic. We drew on a large sample of men enrolled in the Cebu Longitudinal Health and Nutrition Survey (CLHNS; https://cebu.cpc.unc.edu/), which is an ongoing birth cohort study that began in Metropolitan Cebu (Philippines) in 1983–84 [41]. We used socio-demographic and paternal caregiving data collected prior to the pandemic in 2009 (wave 1: fathers’ age range, 25.00–26.63 years) and 2014 (wave 2: ages 29.63–31.34 years) and a third wave post-pandemic in 2022–23 (wave 3, ages 38.50–40.25 years). Our main focus is on within-individual change in paternal caregiving for men with co-residential young children (N = 307) from the pre-pandemic (wave 2; 2014) to post-pandemic period (wave 3; 2022). We focused on fathers’ overall (total) caregiving and their time spent in routine (intensive), recreational, and educational caregiving tasks. We leveraged this large longitudinal dataset to facilitate comparisons with the existing (largely cross-sectional) literature in this area and to allow for examination of temporal relations between key predictors and paternal caregiving time.

We tested the hypothesis that fathers’ caregiving time would be higher post-pandemic than pre-pandemic. To do this, we first included data ranging from 2009 to 2022 in order to characterize the patterning of fathers’ caregiving over time in this setting, where father involvement is relatively understudied compared to Euro-American contexts. We then tested this hypothesis in our main analyses, comparing wave 2 (2014) and wave 3 (2022−23) caregiving data for the men in our within-individual change analyses (N = 307). Next, we drew on key paternal characteristics previously shown to predict paternal caregiving in Cebu (educational attainment; employment status) for our second hypothesis [35,38,42]. We tested the hypothesis that fathers’ educational attainment and employment status would be associated with different patterns of paternal care pre-to-post pandemic. We specifically tested whether better-educated fathers and under-employed men, respectively, would show larger increases in caregiving pre-to-post pandemic than less-educated fathers and fully-employed men, respectively.

## Materials and methods

### Data and participants

Data are from the Cebu Longitudinal Health and Nutrition Survey (CLHNS), an ongoing, population representative birth-cohort study of infants born in 1983−84 in the Metro Cebu region of the Philippines. The singleton infants born as part of this cohort study have been followed at many waves of data collection across childhood and adulthood [41]. For full information on CLHNS sample design and methodologies, see [35,41,43]. Attrition rates during the early years of the project ranged between 9% and 11% and have declined to ∼5% in the adult surveys, with a majority of the attrition resulting from the sample migrating out of Metro Cebu [44]. We report descriptive statistics for the main analytical sample in Table 1 and the entire sample in S1 Table.

**Table 1:** Key Descriptive Statistics for Fathers in the Within-individual Change Analyses.

Variables	*M*	*SD*
Age wave 2 (years)	30.52	0.35
Age wave 3 (years)	39.26	0.34
Average age of co-resident children wave 2 (years)	4.00	2.10
Average age of co-resident children wave 3 (years)	7.90	2.55
Number of co-resident children wave 2 (less than 13 years old)	2.10	1.03
Number of co-resident children wave 3 (less than 13 years old)	2.00	1.10
Married/cohabiting wave 2 (% yes)	99.02	–
Married/cohabiting wave 3 (% yes)	93.49	–
Fully employed wave 2 (% yes)	72.31	–
Fully employed wave 3 (% yes)	78.18	–
Less than high school diploma (wave 3) (% yes)	35.18	–
High school diploma (wave 3) (% yes)	54.07	–
College degree or more (wave 3) (% yes)	10.75	–
Total weekly caregiving time wave 2 (hours)	48.16	32.53
Total weekly caregiving time wave 3 (hours)	37.75	32.21
Change in total caregiving time, wave 2 to wave 3 (hours)	−10.41	39.82

*Note.* N = 307. *M* = Mean; *SD =* Standard Deviation.

Socioeconomic, demographic, and behavioral data were collected during in-home interviews administered by Cebuano-speaking interviewers [41,43]. In the present analysis, we defined men as fathers if they reported having biological, adopted, or step-children. Among men identifying as fathers, > 98% reported having at least one biological child. We defined men as partnered if they reported being legally married or cohabitating; we included partnering status as a covariate. All data were collected with written informed consent and ethical approval from Northwestern University (Evanston, IL) and the University of San Carlos (Cebu City, Philippines). Data collections for the present study took place between the following dates: wave 1 (July 17, 2009 to January 3, 2010); wave 2 (December 6, 2013 to October 13, 2014); wave 3 (October 4, 2022 to October 28, 2023).

### Analytical sample

For our main analyses, which focused on within-individual change through time, we restricted our analyses to co-residential fathers who had full data from wave 2 (2014) and wave 3 (2022−23) in order to compare men to themselves. However, an additional consideration in this regard is that men who were already fathers in 2014, in their early 30s, might only have relatively older children (e.g., > 12 years old) at wave 2 or, particularly, by wave 3. These changing demographics within men’s families through time could confound pre- vs. post-pandemic comparisons. Thus, we limited our within-individual change models to men who had at least one co-residential child who was less than 13 years old at both waves 2 and 3 (N = 307) to help enhance the comparability of men’s pre- vs. post-pandemic family conditions. For this analytical sample (N = 307), fathers had an average of 2.10 children <13 years old (1.03 SD) at wave 2 and 2.00 children <13 years old (1.10 SD) at wave 3.

In our analyses focusing on paternal care across the three waves of the study, we included men who were fathers residing with at least one child <13 years old at waves 1, 2 or 3 and who had complete, necessary demographic and paternal caregiving data for the pertinent wave. In wave 1 (2009), there were 384 eligible co-residential fathers. In wave 2 (2014), there were 465 eligible co-residential fathers. Finally, in wave 3 (2022−23), there were 437 eligible co-residential fathers. Among the total of 649 fathers included, 195 men had data across all three waves, and 442 men had data from at least two waves.

### Dependent variables

#### Paternal caregiving data.

At each wave, fathers reported the amount of time (in hours and minutes) they had allocated in the past week to a list of 20 paternal caregiving behaviors informed by a large-scale survey on fathering and caregiving in the Philippines [42]. The list included the following: preparing children’s meals, feeding them, watching over them, playing, singing/dancing, reading, telling stories, watching television with them, listening to the radio with them, taking them on walks or outings, bathing them, attending to toilet needs or training, dressing or grooming them, putting them to sleep, bringing them to or from to school, and helping them with schoolwork [35]. The domains of caregiving are not mutually exclusive, and thus some behaviors could co-occur. Following Rosenbaum et al. [38], we defined caregiving tasks as follows: ‘routine’ tasks as: bathing, grooming, helping with toilet needs, feeding, and putting to bed; ‘recreational’ tasks as: playing, going for walks/outings, singing/dancing, and exchanging stories; ‘educational’ tasks as: reading, taking children to school, and helping with schoolwork. For these weekly caregiving reports at each wave, we do not have complementary data on maternal or alloparental caregiving time. We also do not have reporting from mothers or another household member on fathers’ weekly caregiving time (in hours/minutes). However, during wave 2 (2014), mothers were also interviewed and asked about fathers’ participation in these caregiving tasks (yes/no). We have previously shown correspondence between these maternal reports of fathers’ involvement in caregiving tasks and fathers’ self-reported caregiving time [38]. We analyzed total, routine, recreational, and educational caregiving time as hours of care and calculated within-individual change in hours for the pre-pandemic (2014 minus 2009) and pre-to-post-pandemic (2022 minus 2014) periods.

### Key independent variables

#### Time period.

We treated time period as a categorical variable denoting the survey wave or as pre-pandemic vs. pre-to-post-pandemic periods for the within-individual change analyses.

#### Educational attainment.

We categorized fathers into three categories for educational attainment: less than a high school diploma, high school graduate, and college graduate or higher degree.

#### Employment status.

At each wave, we defined men as partially employed or unemployed (partially/unemployed) if they reported that they were not working or working less than full time (<40 hours per week); men who were working full time were categorized as fully employed.

### Covariates

We included men’s marital/cohabiting status, their number of co-resident children (less than 13 years old), and the average age of their co-resident children (less than 13 years old) as covariates. For models focused on educational care, we included their number of elementary school-aged children (ages 5–12). In each model, we also included all of the key independent variables listed above as predictors.

### Statistical analyses

We conducted all statistical analyses using Stata v. 18.0 (Stata Corporation). Because the count data for fathers’ caregiving time were right-skewed and over-dispersed in each wave, we used mixed-effects negative binomial regression with maximum-likelihood estimation to analyze caregiving time by wave (Stata’s ‘menbreg’ command). We used ordinary least squares regression to predict fathers’ within-individual changes in caregiving time. Following significant results for the within-individual change models, we ran follow-up models using linear mixed models to compare patterns of change for pre-pandemic (2014 minus 2009) and pre-to-post-pandemic (2022 minus 2014) time periods based on fathers’ characteristics. For models including mixed effects, we included a random intercept effect for each individual participant to account for the structure of the data, with individuals having multiple observations collected across waves 1–3.

We first tested for differences in fathers’ caregiving time by survey wave, including for waves 1, 2, and 3 for the overall sample. Next, we conducted two sets of within-individual change analyses for fathers who had at least one co-residential child who was less than 13 years old at both waves 2 and 3. First, we focused on change from wave 2 to wave 3. Next, to evaluate individual differences for changes in care, we drew on several characteristics of fathers, namely: men’s educational attainment and employment status. We calculated within-individual change in these paternal characteristics (e.g., did a father go from being partially/unemployed to fully employed between waves 2 and 3) and tested whether they predicted concomitant change in fathers’ caregiving time. In our follow-up models (see above), we used these paternal characteristics in moderation analyses. Given that these moderation analyses involved several additional statistical tests and were exploratory in nature, we applied the Benjamini–Hochberg procedure for multiple testing correction [45,46]. We generated the figures using predictive margins (adjusted predictions) using Stata’s ‘margins’ and ‘marginsplot’ commands. We evaluated statistical significance at *p* < .05.

## Results

### Variation and change in fathers’ caregiving

**Fig 1:**
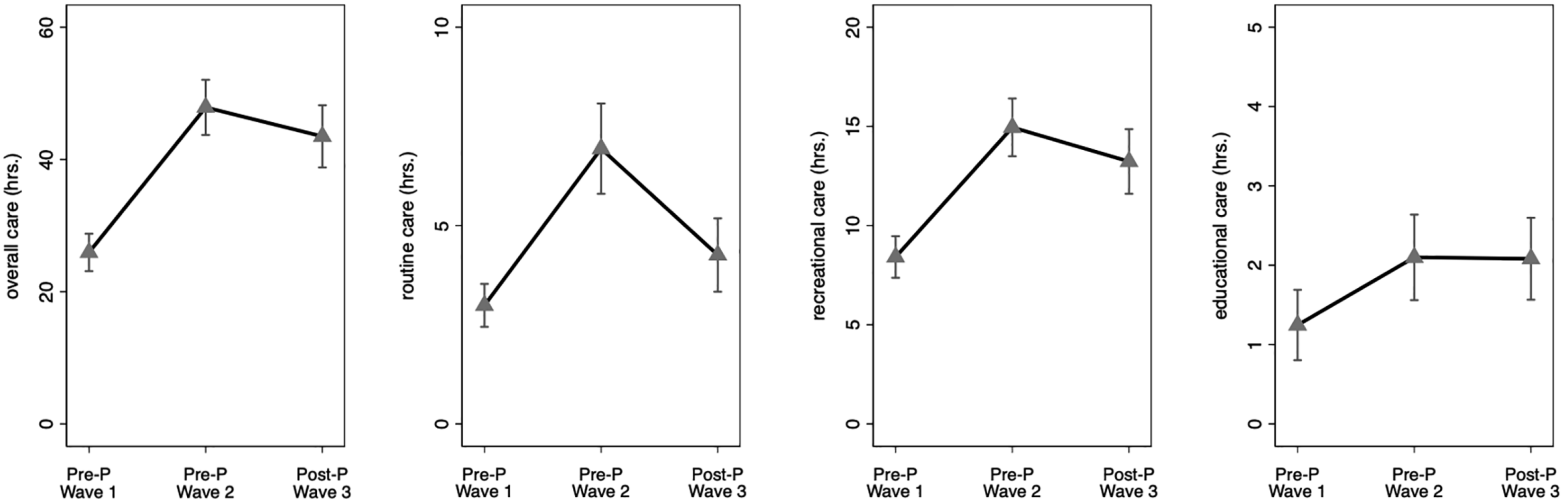
Temporal patterning of fathers’ overall, routine, recreational, and educational caregiving time in the past week for the entire sample (N = 649; Obs. = 1286), by pre-pandemic (Pre-P) and post-pandemic (Post-P) waves. The plots were derived using predictive margins (adjusted predictions), following the models predicting fathers’ caregiving time (hours) in S2 Table. Error bars indicate 95% confidence intervals. Wave 1: 2009; Wave 2: 2014; Wave 3: 2022−23. The y-axis in each panel ranges ~2 standard deviations of the respective caregiving variable.

To test our first hypothesis and to characterize the long-term, temporal patterning of paternal care in this setting, we first tested for differences in fathers’ caregiving time between waves using the post-pandemic time point (wave 3) as the comparison. Adjusting for key variables (S2 Table), we found that fathers reported greater time spent in overall, routine, recreational, and educational care during the post-pandemic wave compared to wave 1 (i.e., 2009; all *p* < .05). In contrast, fathers reported comparable engagement in overall, recreational, and educational care post-pandemic relative to wave 2 (i.e., 2014; all *p* > .1; Table S2; Fig 1), and fathers reported less time in routine care post-pandemic than in wave 2 (*p* < .001).

**Fig 2:**
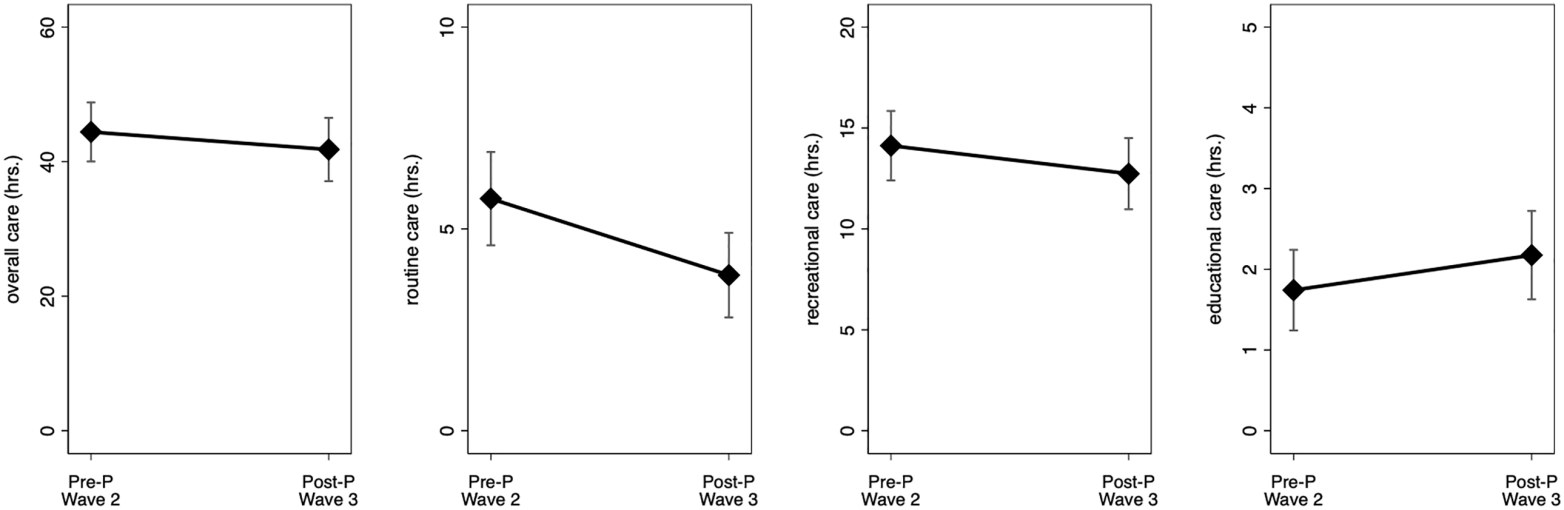
Within-individual patterns for fathers’ overall, routine, recreational, and educational caregiving time in the past week from pre-pandemic (Pre-P) wave 2 to post-pandemic (Post-P) wave 3 (N = 307; Obs. = 614). This figure illustrates within-individual caregiving patterns for fathers with young co-resident children at both waves 2 and 3. The plots were derived using predictive margins (adjusted predictions), following the models predicting fathers’ caregiving time (hours) in Table 2. Error bars indicate 95% confidence intervals. Wave 2: 2014; Wave 3: 2022−23. The y-axis in each panel ranges ~2 standard deviations of the respective caregiving variable.

In our main analyses specifically focusing on fathers’ within-individual changes in caregiving from pre-to-post pandemic, we then compared men’s overall, routine, recreational, and educational caregiving time at wave 3 (2022) to their own values at wave 2 (2014) for fathers who had children less than 13 years old in each wave. Fathers reported similar levels of overall, recreational, and educational care pre-pandemic (wave 2) compared to post-pandemic (all *p* > .2; Table 2; Fig 2). However, fathers reported greater involvement in routine care pre-pandemic compared to post-pandemic (*p* = .01; Table 2; Fig 2).

**Table 2:** Predicting Fathers’ Within-individual Patterns in Caregiving Time Between Pre-pandemic (Wave 2) and Post-pandemic (Wave 3) Waves (N = 307; Obs. = 614).

	Overall caregiving			Routine caregiving			Recreational caregiving			Educational caregiving		
Predictor	*IRR*	*SE*	*p*	*IRR*	*SE*	*p*	*IRR*	*SE*	*p*	*IRR*	*SE*	*p*
Pre-pandemic (wave 2)	1.06	0.09	.470	1.49	0.23	.010	1.11	0.11	.277	0.80	0.15	.228
Married	0.95	0.16	.777	0.46	0.16	.026	1.08	0.22	.702	1.64	0.67	.224
High school	1.25	0.09	.002	1.59	0.24	.002	1.36	0.12	.001	1.65	0.29	.004
College or greater	1.40	0.16	.004	1.59	0.39	.059	1.88	0.28	<.001	2.02	0.56	.012
Fully employed	0.66	0.05	<.001	0.47	0.07	<.001	0.75	0.07	.001	0.41	0.07	<.001
Number of co-residential school-aged children (5-12 years old)	--	--	--	--	--	--	--	--	--	1.34	0.13	.003
Number of co-residential children <13 years old	1.04	0.03	.252	1.17	0.08	.016	0.98	0.04	.600	--	--	--
Average child age (years)	0.95	0.01	.001	0.80	0.02	<.001	0.95	0.02	.002	1.05	0.04	.150

*Note. N* = 307; Observations = 614. *IRR* = incident rate ratio. *SE* = Standard error. Reference groups for categorical variables: the post-pandemic period (wave 3); men with less than a high school diploma; men who were partially employed or unemployed; men who were not married/cohabiting.

### Within-individual change in fathers’ caregiving pre- to post-pandemic by key predictors

We then tested whether key variables predicted fathers’ within-individual change scores in caregiving time, from the pre-pandemic (wave 2) to post-pandemic (wave 3). Fathers with a high school diploma or college degree, respectively, showed greater declines pre-to-post pandemic in routine caregiving than those with less than a high school diploma (both *p* < .05; Table 3). Overall caregiving time also declined more among fathers with a high school diploma compared to the change for men with less than a high school diploma (*p* < .05; Table 3).

**Table 3:** Predicting change in fathers’ caregiving time between time periods (N = 307; Obs. = 614).

	Change in overall caregiving			Change in routine caregiving			Change in educational caregiving		
Predictor	*b*	95% CI	*p*	*b*	95% CI	*p*	*b*	95% CI	*p*
High school	−9.60	−19.18, −0.02	.049	−3.61	−5.73, −1.50	.001	−0.03	−1.06, 1.00	.957
College or greater	−4.16	−20.29, 11.97	.612	−3.67	−7.23, −0.11	.044	0.80	−0.94, 2.54	.365
Fully employed to partially/unemployed	24.92	7.49, 42.35	.005	4.72	0.87, 8.57	.016	3.70	1.82, 5.57	<.001
Partially/unemployed (both waves)	25.36	8.18, 42.53	.004	8.06	4.26, 11.85	<.001	1.92	0.07, 3.77	.042
Fully employed (both waves)	15.64	3.40, 27.88	.012	3.77	1.06, 6.47	.006	1.69	0.37, 3.00	.012
Change in number of co-residential children <13 years old	2.98	−0.32, 6.29	.077	0.42	−0.31, 1.15	.260	0.26	−0.09, 0.62	.147
Change in average child age (years)	−1.23	−2.47, 0.01	.051	−0.50	−0.78, −0.23	<.001	0.16	0.03, 0.29	.018

Note. N = 307; Observations = 614. CI = Confidence interval. Reference groups for categorical variables: men with less than a high school diploma; Reference groups for categorical variables: men with less than a high school diploma; men who went from being partially/unemployed (wave 2) to fully employed (wave 3). Models adjust for change in the number of co-residential children (under 13 years old). We did not adjust for changes in marital/cohabiting status in these models because a strong majority of fathers remained married/cohabiting at both waves (93.49%).

**Fig 3:**
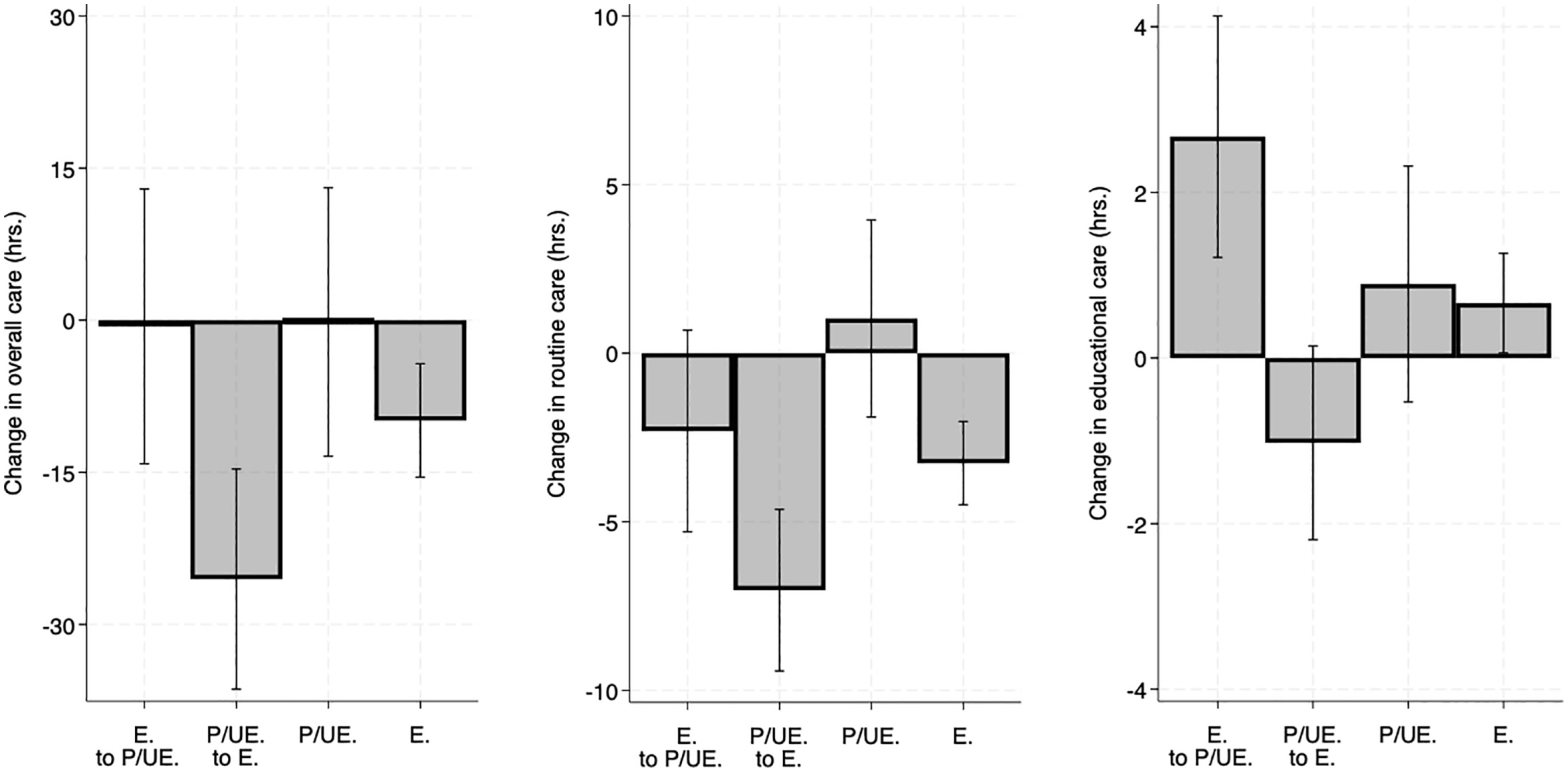
Fathers’ pre-to-post pandemic, within-individual change in overall, routine, and educational caregiving time, stratified by employment status (N = 307). The plots were derived using predictive margins (adjusted predictions), following the models predicting fathers’ change in caregiving time (hours) in Table 3. Error bars indicate 95% confidence intervals. E = fully employed; P/UE = partially employed or unemployed. Categories labeled only “P/UE” or “E” indicate fathers whose employment status was stable in that category between waves 2 and 3. The y-axis in each panel ranges ~2 standard deviations of the respective change in caregiving variable.

Fathers’ change in employment status was the most consistent predictor of change in care pre-to-post pandemic. As shown in Fig 3, fathers who went from partially/unemployed pre-pandemic to fully employed post-pandemic (the comparison group) showed larger declines in overall, routine, and educational care compared to other groups of fathers, including those who became partially/unemployed post-pandemic and those whose employment status remained stable (all *p* < .05; Table 3). Fathers’ changes in recreational care did not significantly differ based on these key predictors (see S3 Table).

### Follow-up models: comparing change in care prior to the pandemic (wave 1 to 2) and pre-to-post pandemic change (wave 2 to 3)

Given the within-individual change results for educational attainment and employment status, we then ran follow-up models comparing patterns of change for pre-pandemic (wave 1 to wave 2) and pre-to-post-pandemic time (wave 2 to wave 3) periods based on those characteristics of fathers. The goal of these models was to test whether the associations between such characteristics (e.g., employment shifts) and change in care were significantly different pre- vs. pre-to-post-pandemic. As noted in the analytic plan, we applied the Benjamini–Hochberg procedure when conducting these exploratory analyses, so as to decrease the false discovery rate; therefore, p-values reported in S4 Table are Benjamini–Hochberg adjusted.

#### Educational attainment.

**Fig 4:**
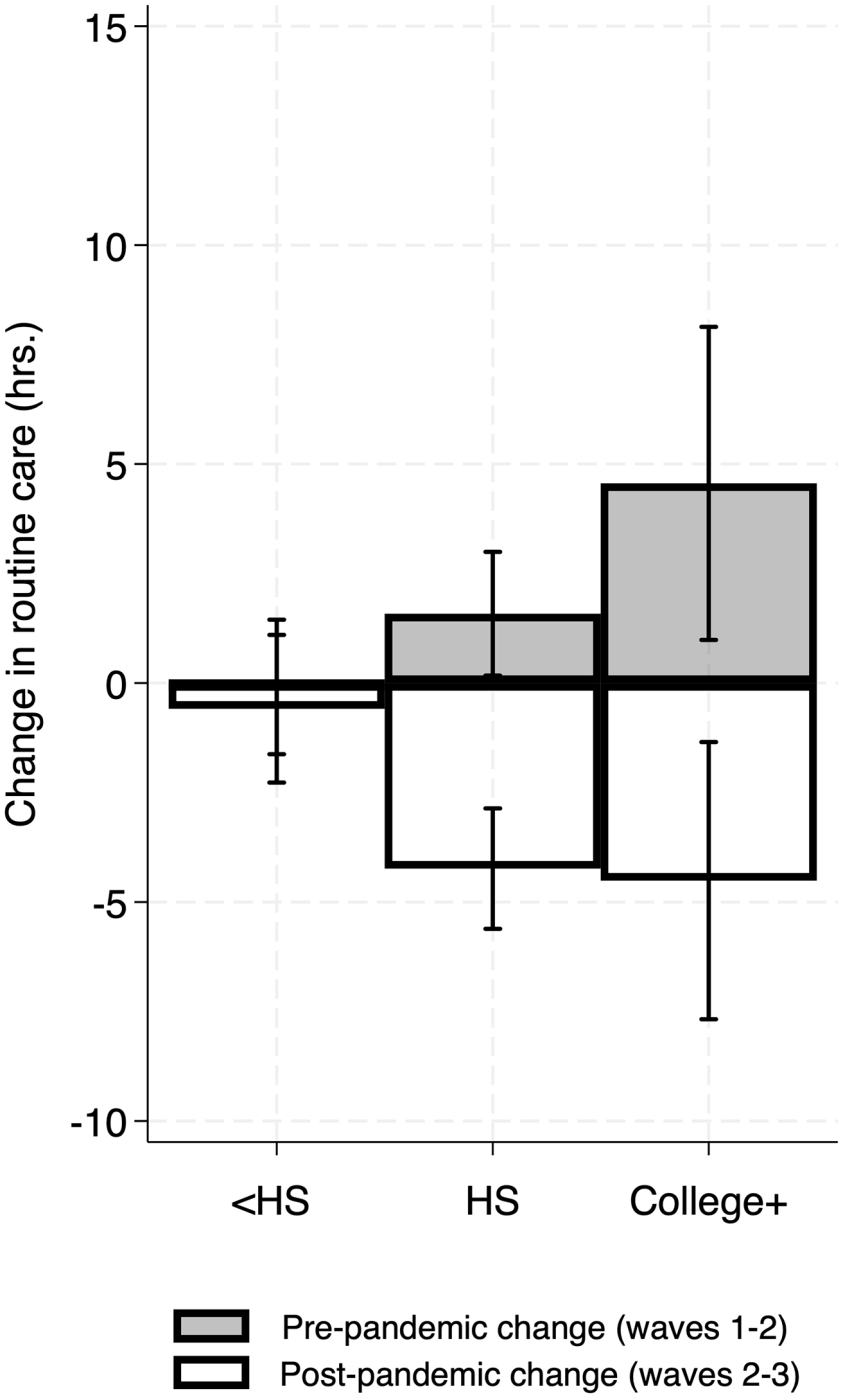
Fathers’ within-individual change in routine caregiving time by pre-pandemic (Pre-P) and pre-to-post-pandemic (Post-P) time periods, stratified by education level (N = 421; Obs. = 616). The plots were derived using predictive margins (adjusted predictions), following the models predicting fathers’ change in caregiving time (hours) in S4 Table. Error bars indicate 95% confidence intervals. Pre-pandemic period: change from 2009 to 2014; Post-pandemic period: change from 2014 to 2022−23. HS = high school; College+ = college degree or higher. The y-axis in each panel ranges ~2 standard deviations of the respective change in caregiving variable.

For routine care, there were significant interactions for (time period x fathers’ educational level; adjusted p-values both *p* < .01; S4 Table). Specifically, better-educated fathers showed larger discrepancies for change in caregiving time between the pre-pandemic period and the pre-to-post-pandemic period, compared to differences in caregiving time for fathers without a high school diploma between the two periods (see Fig 4). As shown in Fig 4, better-educated fathers’ routine caregiving time declined more pre-to-post pandemic, on average, for routine care, compared to fathers with less than a high school diploma. In contrast, during the pre-pandemic period, better-educated fathers’ care time increased comparatively more.

#### Employment status.

**Fig 5:**
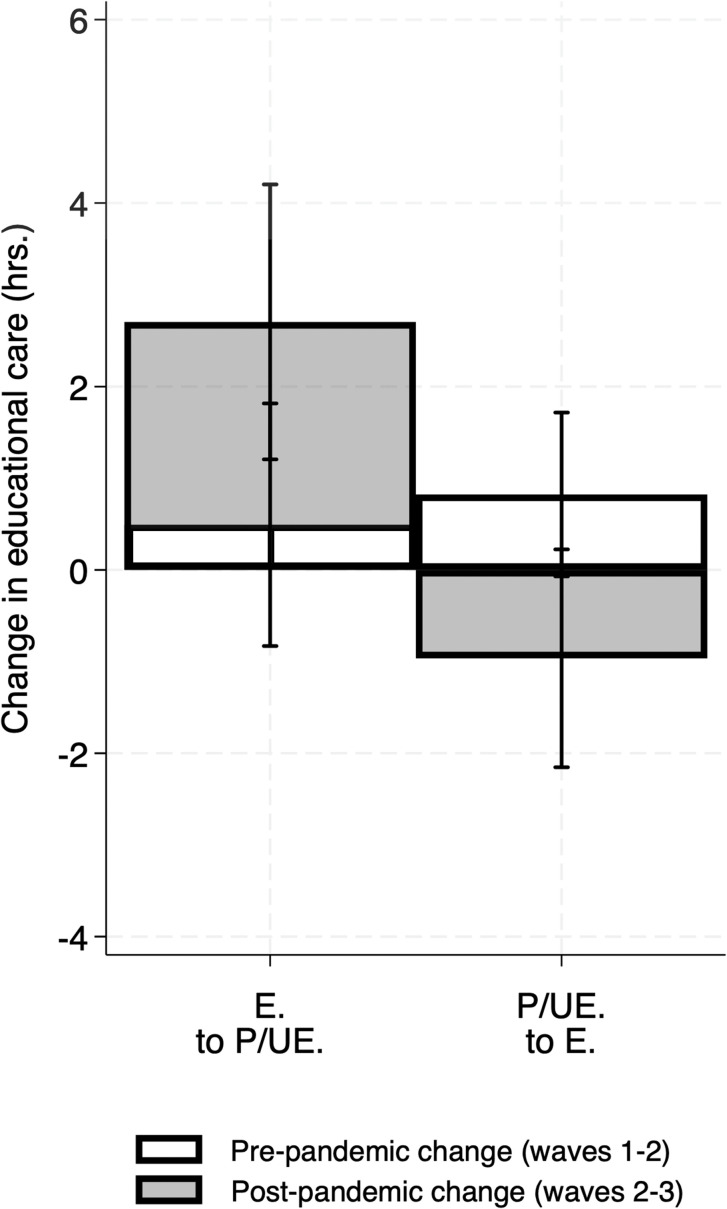
Fathers’ within-individual change in overall and educational caregiving time by pre-pandemic (Pre-P) and pre-to-post-pandemic (Post-P) time periods, stratified by employment status (N = 421; Obs. = 616). The plots were derived using predictive margins (adjusted predictions), following the models predicting fathers’ change in caregiving time (hours) in S4 Table. Error bars indicate 95% confidence intervals. Pre-pandemic period: change from 2009 to 2014; Post-pandemic period: change from 2014 to 2022-23. E = fully employed; P/UE = partially employed or unemployed. The y-axis in each panel ranges ~2 standard deviations of the respective change in caregiving variable.

For both educational care and routine care, there were significant interactions for (time period x fathers’ employment status; adjusted p-values both *p* < .05; S4 Table). Specifically, as shown in Fig 5, the differences for changes in educational care (pre-pandemic vs. pre-to-post-pandemic) for fathers who went from partially/unemployed to fully employed were significantly smaller than the equivalent differences for changes in care for fathers who went from fully employed to partially/unemployed. For routine care, fathers who went from fully employed to partially/unemployed showed modest increases in care during both periods (pre-pandemic vs. pre-to-post-pandemic). In contrast, fathers who went from partially/unemployed to fully employed showed relatively large decreases in routine care in the pre-to-post-pandemic period.

## Discussion

In this prospective, longitudinal analysis of Filipino fathers’ caregiving time, we found that fathers’ caregiving time decreased pre-to-post pandemic, on average, in terms of their time devoted to routine caregiving. In contrast, fathers maintained their time spent in overall, recreational, and education-related care activities from pre-to-post-pandemic. Shifting family dynamics and demographics across the study period are one potential driver of the patterns for routine caregiving, although we adjusted our models for relevant family covariates, such as number of co-resident children (<13 years old) in the household and the average age of those co-residential children. Fathers’ transition from their early 30s (pre-pandemic) to their late 30s (post-pandemic) corresponded with decreases in their likelihood of having infants and other young children requiring intensive care during the studied time frame [47]. Nonetheless, compared to themselves pre-pandemic (wave 2; 2014), fathers showed particularly meaningful differences for post-pandemic (wave 3; 2022) routine caregiving (effect size: 0.45 SDs). In total, the average patterns of change do not provide evidence for the idea that the prolonged pandemic lockdown period in the Philippines helped facilitate increased paternal caregiving time post-pandemic, on average, although they do suggest that fathers largely maintained their caregiving in multiple domains pre-to-post pandemic.

Because pandemic-related increases in father-child time and paternal caregiving differed by family context [19,24–26], we also tested whether key paternal characteristics were linked with within-individual changes in paternal care pre-to-post pandemic. We drew on established predictors of paternal caregiving in this setting [35,38], hypothesizing that specific sub-groups of fathers— e.g., well-educated men— might have been more likely to increase their caregiving during the pandemic and sustain those patterns thereafter. We found some modest support for this hypothesis, specifically related to fathers’ employment.

Specifically, in the pre-to-post-pandemic period, men going from partially/unemployed to fully employed meaningfully decreased their overall, routine, and educational caregiving. In contrast, those going from fully employed to partially/unemployed maintained their overall caregiving and increased their educational caregiving, on average, and also showed a comparatively smaller decline in their routine caregiving. For these domains of care, men who were partially/unemployed across the two waves also had broadly similar patterns. Our models comparing patterns of change pre-pandemic (wave 1 to wave 2) versus pre-to-post pandemic (wave 2–3) also demonstrate that the pre-to-post pandemic patterns of routine and educational caregiving and employment change are distinct from how they changed together pre-pandemic (wave 1 to wave 2). For example, in the pre-pandemic period (wave 1 to wave 2) men tended to increase their educational caregiving time, regardless of their employment shifts (see Fig 5); however, in the pre-to-post pandemic period men who went from fully employed to partially/unemployed substantially increased their educational care (0.59 SDs), on average, whereas men transitioning to full employment tended to decrease such care (−0.26 SDs). This approach provides some evidence that aligns with the idea that these patterns may have been shaped by dynamics of the pandemic. However, a number of other explanations are likewise plausible, including that fathers’ aging (and associated shifts in their orientation to dimensions of fathering) and changing family demographics help to explain the results, and we cannot rule them out.

Our results document links between men’s employment status shortly after the end of the pandemic lockdown period and their involvement in childcare. Thus, they have some broad complementarity with existing longitudinal research on fathers’ caregiving and pandemic-period employment dynamics in Euro-American contexts [24,25]. For example, U.S. fathers working exclusively from home or working only part time during the pandemic reported greater childcare involvement than their peers working outside the home and full time, respectively [25]. These past results and our findings are observational and therefore cannot speak to causal pathways. However, they generally align with the conceptual possibility that fathers’ employment dynamics, such as through partial/unemployment or working from home, during and following the pandemic helped shape their availability to their children and thereby could have served as a potential influence on their caregiving involvement across a range of societal settings. In addition to the effects of the pandemic and the prolonged shutdown period in the Philippines, there were also other significant concurrent shocks to this region, particularly Super Typhoon Rai in 2021, which substantially affected public health, infrastructure, and livelihoods [48]. This natural disaster could likewise have affected men’s employment dynamics, including in combination with or separate from the pandemic.

For the other key predictor related to within-individual change in paternal caregiving time— fathers’ educational attainment— we found mixed results. Consistent with our predictions, we found that college-educated fathers engaged in greater overall, routine, and recreational caregiving time prior to the pandemic (wave 2; 2014) and that their higher overall involvement than less-educated fathers persisted after the pandemic. However, we also observed a significant effect of fathers’ educational attainment on within-individual change in routine caregiving time pre-to-post pandemic but in an unanticipated direction. Better-educated fathers experienced post-pandemic decreases in routine caregiving time, on average, that exceeded the caregiving declines for less-educated fathers. Contrasting with this result, we had anticipated that college-educated fathers might increase or maintain their caregiving time post-pandemic, as they would be more likely to have professional or office jobs with options to work from home as well as the necessary resources (e.g., reliable internet access) for such roles, compared to those employed in manual or service labor. College-educated fathers’ caregiving time increased much more pre-pandemic, compared to less-educated fathers, and they engaged in more absolute caregiving time pre-pandemic (wave 2; 2014) and post-pandemic (wave 3; 2022). Nonetheless, college-educated fathers’ temporal patterns of caregiving through time, particularly for routine care, are not consistent with the idea that pandemic conditions facilitated enhanced caregiving involvement for this sub-group of fathers.

### Limitations

A main limitation of our analysis is that we do not have data for the study during the pandemic lockdown period. Thus, unlike some of the few other longitudinal analyses in this area, we cannot analyze potentially shorter-term shifts in fathers’ caregiving dynamics at the onset of the pandemic and as it progressed [24,25]. Relatedly, given that our study participants are all birth cohort members and are thus approximately the same age, it is not possible for us to fully disentangle the effect of the pandemic from the effect of advancing age. This is particularly the case given that the pre- and post-pandemic measures were obtained about 8 years apart, which is not a trivial change in men’s adult life course and family demographics. Compensating somewhat for these limitations, we structured our analyses to focus on fathers with children less than 13 years old both pre- and post-pandemic and adjusted our analyses for the number of co-residential children less than 13 years and the average age of men’s co-residential children. Moreover, our research design does enable us to provide timely insights on post-pandemic patterns of paternal care as families emerged from a long lockdown period and attempted to return to normalcy. An additional strength of our longitudinal design is that it relies on prospectively collected data on paternal caregiving time from the pre-pandemic waves, rather than retrospective reports.

An additional limitation is that our paternal caregiving data come from a single reporter. However, prior research from this setting using the wave 2 (2014) data when mothers were also interviewed showed correspondence between paternal and maternal reports of fathers’ involvement in caregiving tasks [38]. A related, final limitation is that we do not also have equivalent data on maternal or alloparental caregiving time. Such data would help us to more fully assess the extent to which the post-pandemic declines in fathers’ caregiving time represent a further, emergent, gendered imbalance in caregiving within families in this setting, including for intensive tasks, such as routine caregiving [8,10,11].

## Conclusion

There is much interest in shifts in childcare and domestic responsibilities in different-gender parent households that may have emerged during and after the pandemic period. In part, this interest derives from the notion that fathers’ increased time spent with their children during the pandemic could facilitate lasting effects on their caregiving involvement and enhance caregiving equity in families with different-gender parents. In this way, the pandemic is a natural-occurring event that can inform our understanding of policies that aim to increase fathers’ time with their children as a means to enhance long-term paternal involvement [12]. Few studies have pre-to-post pandemic, prospective longitudinal data on fathers’ contributions in such families, including in global contexts outside of Euro-American societies [1]. Drawing on longitudinal data collected over almost a decade in the Philippines, our study contributes to this area by showing that Filipino fathers’ routine caregiving time declined post-pandemic, compared to pre-pandemic levels, while their care in the other domains we tested were relatively stable, on average. We also found evidence that shifts in men’s employment status pre-to-post pandemic were associated with meaningful concurrent changes in their caregiving, complementing results from other societal settings that highlighted the relevance of parental work dynamics to fathers’ caregiving during the pandemic. In sum, our results provide limited support for the hypothesis that the pandemic had lasting, amplifying effects on paternal caregiving contributions in this setting.

## Supporting information

S1 Table Key Descriptive Statistics for the Entire Sample. (DOCX)

S2 Table Predicting Fathers’ Caregiving Time Between Waves in 2009, 2014, and 2022. (DOCX)

S3 Table Predicting Within-Individual Change in Fathers’ Recreational Caregiving Time from Pre-pandemic (Wave 2) to Post-Pandemic (Wave 3). (DOCX)

S4 Table Predicting Change in Fathers’ Caregiving Time by Time Period (Pre-pandemic vs. Pre-to-post-pandemic): Moderation by Employment Status and Education Level. (DOCX)

S1 File CLHNS wave by wave paternal care data. (XLS)

S2 File CLHNS within individual change paternal care data. (XLS)

S3 File Gettler et al PLOS One codebook. (DOCX)
