# Supplementary material for: Fathers’ caregiving time before and after the COVID-19 pandemic
Source: PLoS One. 2026 Mar 16;21(3):e0343636. doi: 10.1371/journal.pone.0343636 (PMC12991276; doi:10.1371/journal.pone.0343636)
Supplement: S1 Table — (DOCX) [file pone.0343636.s001.docx]

| **S1 Table. Key Descriptive Statistics for the Entire Sample** | | |
| --- | --- | --- |
| Variables | *M* | *SD* |
| Age wave 1 (years) | 25.99 | 0.30 |
| Age wave 2 (years) | 30.52 | 0.34 |
| Age wave 3 (years) | 39.27 | 0.34 |
| Average age of co-resident children wave 1 (years) | 2.16 | 1.67 |
| Average age of co-resident children wave 2 (years) | 4.39 | 2.24 |
| Average age of co-resident children wave 3 (years) | 7.27 | 2.89 |
| Number of co-resident children wave 1 (less than 13 years old) | 1.60 | 0.79 |
| Number of co-resident children wave 2 (less than 13 years old) | 2.02 | 1.00 |
| Number of co-resident children wave 3 (less than 13 years old) | 1.91 | 1.05 |
| Married/cohabiting wave 1 (% yes) | 99.48 | - |
| Married/cohabiting wave 2 (% yes) | 97.20 | - |
| Married/cohabiting wave 3 (% yes) | 93.82 | - |
| Fully employed wave 1 (% yes) | 53.91 | - |
| Fully employed wave 2 (% yes) | 68.82 | - |
| Fully employed wave 3 (% yes) | 76.66 | - |
| Less than high school diploma (wave 3) (% yes) | 33.64 | - |
| High school diploma (wave 3) (% yes) | 53.09 | - |
| College degree or more (wave 3) (% yes) | 13.27 | - |
| Total weekly caregiving time wave 1 (hours) | 28.57 | 33.32 |
| Total weekly caregiving time wave 2 (hours) | 46.69 | 32.25 |
| Total weekly caregiving time wave 3 (hours) | 39.97 | 36.36 |
| Change in total caregiving time, wave 1 to wave 2 (hours) | 20.24 | 42.34 |
| Change in total caregiving time, wave 2 to wave 3 (hours) | -7.03 | 40.73 |

*Note. M* = Mean; *SD* = Standard Deviation. Total N = 649. Sample sizes by time period: wave 1, N = 384; wave 2, N = 465; wave 3, N = 437. Sample sizes for change data: wave 1 to wave 2, N = 384; wave 2 to wave 3, N = 449.
