# Supplementary material for: Fathers’ caregiving time before and after the COVID-19 pandemic
Source: PLoS One. 2026 Mar 16;21(3):e0343636. doi: 10.1371/journal.pone.0343636 (PMC12991276; doi:10.1371/journal.pone.0343636)
Supplement: S2 Table — (DOCX) [file pone.0343636.s002.docx]

| **S2 Table. Predicting fathers' caregiving time between waves in 2009, 2014, and 2022 (N = 649; Obs. = 1286)** | | | | | | | | | | | | | |
| --- | --- | --- | --- | --- | --- | --- | --- | --- | --- | --- | --- | --- | --- |
|  | Overall  caregiving | | | Routine  caregiving | | | Recreational caregiving | | | Educational  caregiving | | | |
| Predictor | IRR | *SE* | *p* | IRR | *SE* | *p* | IRR | *SE* | *p* | IRR | *SE* | *p* |  |
| Wave 1 | 0.60 | 0.05 | < .001 | 0.70 | 0.11 | .020 | 0.64 | 0.06 | < .001 | 0.60 | 0.13 | .017 |  |
| Wave 2 | 1.10 | 0.07 | .156 | 1.63 | 0.19 | < .001 | 1.13 | 0.09 | .106 | 1.01 | 0.16 | .956 |  |
| Married | 1.00 | 0.14 | .980 | 0.64 | 0.16 | .080 | 1.28 | 0.21 | .129 | 1.24 | 0.43 | .529 |  |
| High school | 1.23 | 0.07 | .001 | 1.51 | 0.16 | < .001 | 1.31 | 0.09 | < .001 | 1.62 | 0.25 | .002 |  |
| College or greater | 1.52 | 0.14 | < .001 | 1.98 | 0.33 | < .001 | 1.92 | 0.21 | < .001 | 2.70 | 0.62 | < .001 |  |
| Fully employed | 0.75 | 0.04 | < .001 | 0.59 | 0.06 | < .001 | 0.87 | 0.05 | .032 | 0.53 | 0.07 | < .001 |  |
| Number of co-residential school-aged children (5-12 years old) | -- | -- | -- | -- | -- | -- | -- | -- | -- | 1.48 | 0.14 | < .001 |  |
| Number of co-residential children <13 years old | 1.04 | 0.03 | .129 | 1.19 | 0.06 | .001 | 0.99 | 0.03 | .801 | -- | -- | -- |  |
| Average child age (years) | 0.97 | 0.01 | .008 | 0.85 | 0.02 | < .001 | 0.97 | 0.01 | .015 | 1.11 | 0.04 | .002 |  |

*Note*. N = 649; Observations = 1286. SE = Standard error. IRR = Incidence Rate Ratio. Reference groups for categorical variables: the post-pandemic period (wave 3); men with less than a high school diploma; men who were partially employed or unemployed; men who were not married/cohabiting.
