# Supplementary material for: Fathers’ caregiving time before and after the COVID-19 pandemic
Source: PLoS One. 2026 Mar 16;21(3):e0343636. doi: 10.1371/journal.pone.0343636 (PMC12991276; doi:10.1371/journal.pone.0343636)
Supplement: S3 Table — (DOCX) [file pone.0343636.s003.docx]

| **S3 Table.** **Predicting Within-Individual Change in Fathers' Recreational Caregiving Time from Pre-pandemic (Wave 2) to Post-Pandemic (Wave 3) (N = 307)** | | | |
| --- | --- | --- | --- |
|  | Change in recreational caregiving | | |
| Predictor | *b* | 95% CI | *p* |
| High school | -0.80 | -4.53, 2.93 | 0.673 |
| College or greater | -1.03 | -7.31, 5.25 | 0.747 |
| Fully employed to partially/unemployed | 3.51 | -3.28, 10.29 | 0.310 |
| Partially/unemployed (both waves) | 2.20 | -4.48, 8.88 | 0.518 |
| Fully employed (both waves) | 2.10 | -2.66, 6.87 | 0.386 |
| Change in number of co-residential children <13 years old | -0.16 | -1.45, 1.12 | 0.804 |
| Change in average child age (years) | -0.62 | -1.10, -0.14 | 0.012 |

*Note. N* = 307. CI = Confidence interval. Reference groups for categorical variables: men with less than a high school diploma; Reference groups for categorical variables: men with less than a high school diploma; men who went from being partially/unemployed (wave 2) to fully employed (wave 3). We did not adjust for changes in marital/cohabiting status in these models because a strong majority of fathers remained married/cohabiting at both waves (93.49%).
