# Supplementary material for: Fathers’ caregiving time before and after the COVID-19 pandemic
Source: PLoS One. 2026 Mar 16;21(3):e0343636. doi: 10.1371/journal.pone.0343636 (PMC12991276; doi:10.1371/journal.pone.0343636)
Supplement: S4 Table — (DOCX) [file pone.0343636.s004.docx]

| **S4 Table. Predicting Change in Fathers' Caregiving Time by Time Period (Pre-pandemic vs. Pre-to-post-pandemic): Moderation by Employment Status and Education Level (N = 421; Obs. = 616)** | | | | | | | | | |
| --- | --- | --- | --- | --- | --- | --- | --- | --- | --- |
|  | Change in overall caregiving | | | Change in routine caregiving | | | Change in educational caregiving | | |
|  | *Coef.* | 95% CI | *adjusted p* | *Coef.* | 95% CI | *adjusted p* | *Coef.* | 95% CI | *adjusted p* |
| Pre-pandemic | 23.15 | 7.63, 38.67 | .016 | 3.80 | 0.39, 7.20 | .037 | 1.45 | -0.22, 3.13 | .165 |
| High school | -9.68 | -19.36, 0.00 | .093 | -3.65 | -5.77, -1.52 | .003 | -0.02 | -1.06, 1.03 | .975 |
| College or greater | -5.24 | -21.53, 11.05 | .528 | -3.92 | -7.50, -0.35 | .037 | 0.74 | -1.02, 2.50 | .533 |
| High school x pre-pandemic | 20.86 | 7.47, 34.26 | .016 | 5.32 | 2.38, 8.26 | < .001 | 0.55 | -0.89, 2.00 | .538 |
| College or greater x pre-pandemic | 23.27 | -0.67, 47.22 | .093 | 8.57 | 3.31, 13.83 | .003 | 0.56 | -2.02, 3.15 | .726 |
| Fully employed to partially/unemployed | 25.11 | 7.52, 42.70 | .016 | 4.81 | 0.95, 8.67 | .026 | 3.67 | 1.77, 5.57 | < .001 |
| Partially/unemployed (both waves) | 24.82 | 7.45, 42.19 | .016 | 7.93 | 4.12, 11.74 | < .001 | 1.89 | 0.01, 3.76 | .127 |
| Fully employed (both waves) | 15.43 | 3.05, 27.81 | .032 | 3.72 | 1.01, 6.44 | .015 | 1.66 | 0.33, 3.00 | .049 |
| Employed to partially/unemployed x pre-pandemic | -19.12 | -42.03, 3.79 | .132 | -2.97 | -8.00, 2.06 | .247 | -4.00 | -6.47, -1.53 | .009 |
| Partially/unemployed (both waves) x pre-pandemic | -13.85 | -35.74, 8.05 | .233 | -5.93 | -10.74, -1.13 | .026 | -1.20 | -3.56, 1.16 | .462 |
| Employed (both waves) x pre-pandemic | -15.10 | -31.52, 1.32 | .103 | -4.12 | -7.72, -0.51 | .036 | -1.37 | -3.14, 0.40 | .210 |

*Note.* *N* = 421; Observations = 616. CI = Confidence Interval. Reference groups for categorical variables: the pre-to-post pandemic period (wave 2 to wave 3); men with less than a high school diploma; men who went from being partially/unemployed to fully employed. Models adjust for change in the number of co-residential children (under 13 years old) and change in average child age. We did not adjust for changes in marital/cohabiting status in these models because a strong majority of fathers remained married/cohabiting across waves. For these exploratory follow-up analyses, we applied the Benjamini-Hochberg correction to decrease the false discovery rate; all p values reported in this table are therefore Benjamini-Hochberg adjusted. CIs are unadjusted.
