# Supplementary material for: Fathers’ caregiving time before and after the COVID-19 pandemic
Source: PLoS One. 2026 Mar 16;21(3):e0343636. doi: 10.1371/journal.pone.0343636 (PMC12991276; doi:10.1371/journal.pone.0343636)
Supplement: S3 File — (DOCX) [file pone.0343636.s007.docx]

**Fathers’ caregiving time before and after the COVID-19 pandemic**

*Codebook

**Variables in file “CLHNS wave by wave paternal care data”**

newid: participant identifier

totalcare: hours of total childcare

routine: hours of routine childcare

recreation: hours of recreational childcare

educare: hours of educational childcare

year: year of data collection; 1 = 2009, 2 = 2014, 3 = 2022-23

under13kids: number of co-resident children less than 13 years old

schoolkids: number of elementary school-aged children (ages 5-12)

kidage_avg: average age of co-residential children less than 13 years old

college: educational attainment; 0 = less than a high school diploma; 1 = high school diploma; 2 = college degree or more

fulltime: employment status; 0 = partially employed or unemployed (partially/unemployed); 1 = working full time

married: 0 = not married; 1 = married

inwave23: 1 = fathers with relevant data in both 2014 (wave 2) and 2022-23 (wave 3)

under13wave23: 1 = fathers with at least one co-resident children less than 13 years old in both 2014 (wave 2) and 2022-23 (wave 3)

**Table 2**

We used Stata’s “menbreg” command for mixed-effects negative binomial regression analyses, and we used Stata’s margins command following the analyses to generate figures.

Below we provide the code for the analyses in Table 2:

menbreg totalcare ib3.year married i.college fulltime kidage_avg under13kids if inwave23==1 & year>1 & under13wave23==1 || newid:, irr

menbreg routine ib3.year married i.college fulltime kidage_avg under13kids if inwave23==1 & year>1 & under13wave23==1 || newid:, irr

menbreg recreation ib3.year married i.college fulltime kidage_avg under13kids if inwave23==1 & year>1 & under13wave23==1 || newid:, irr

menbreg educare ib3.year married i.college fulltime kidage_avg under13kids if inwave23==1 & year>1 & under13wave23==1 || newid:, irr

**Supp. Table 2**

menbreg totalcare ib3.year married i.college fulltime under13kids kidage_avg || newid: , irr

menbreg routine ib3.year married i.college fulltime under13kids kidage_avg || newid:, irr

menbreg recreation ib3.year married i.college fulltime under13kids kidage_avg || newid:, irr

menbreg educare ib3.year married i.college fulltime schoolkids kidage_avg || newid:, irr

**Variables in file “CLHNS within individual change paternal care data”**

*Note: in the within-individual change files, we use the prefix “delta” to signify change variables.*

newid: participant identifier

timeperiod: 2 = change between 2009 (wave 1) and 2014 (wave 2); 3= change between 2014 (wave 2) and 2022-23 (wave 3)

college: educational attainment; 0 = less than a high school diploma; 1 = high school diploma; 2 = college degree or more

deltacare: change in hours of total childcare between time periods, e.g., between 2014 (wave 2) and 2022-23 (wave 3)

deltaroutine: change in hours of routine childcare between time periods, e.g., between 2014 (wave 2) and 2022-23 (wave 3)

deltarec: change in hours of recreational childcare between time periods, e.g., between 2014 (wave 2) and 2022-23 (wave 3)

deltaeduc: change in hours of educational childcare between time periods, e.g., between 2014 (wave 2) and 2022-23 (wave 3)

deltafull:

0 = fully employed in earlier wave to partially/unemployed in the next wave

1 = partially/unemployed in earlier wave to fully employed in next wave

2 = partially/unemployed in both waves

3 = fully employed in both waves

inwave23: 1 = fathers with relevant data in both 2014 (wave 2) and 2022-23 (wave 3)

inwave12: 1 = fathers with relevant data in both 2009 (wave 1) and 2014 (wave 2)

deltau13: change in number of co-resident children less than 13 years old between waves

delta_kidage: change in average age of co-residential children less than 13 years old

u13delta3: fathers with at least one co-resident children less than 13 years old in both 2014 (wave 2) and 2022-23 (wave 3)

u13delta2: fathers with at least one co-resident children less than 13 years old in both 2009 (wave 1) and 2014 (wave 2)

**Table 3; Supp. Table 3**

We used Stata’s “reg” command for OLS regression models.

reg deltacare i.college ib1.deltafull deltau13 delta_kidage if inwave23==1 & timeperiod==3 & u13delta3==1

reg deltaroutine i.college ib1.deltafull deltau13 delta_kidage if inwave23==1 & timeperiod==3 & u13delta3==1

reg deltaeduc i.college ib1.deltafull deltau13 delta_kidage if inwave23==1 & timeperiod==3 & u13delta3==1

reg deltarec i.college ib1.deltafull deltau13 delta_kidage if inwave23==1 & timeperiod==3 & u13delta3==1

**Supp. Table 4**

We used Stata’s “mixed” command for linear mixed models.

mixed deltacare ib1.deltafull##ib3.timeperiod i.college##ib3.timeperiod deltau13 delta_kidage if (inwave23==1 & timeperiod ==3 & u13delta3==1) | (inwave12==1 & timeperiod ==2 & u13delta2==1) || newid:

mixed deltaroutine ib1.deltafull##ib3.timeperiod i.college##ib3.timeperiod deltau13 delta_kidage if (inwave23==1 & timeperiod ==3 & u13delta3==1) | (inwave12==1 & timeperiod ==2 & u13delta2==1) || newid:

mixed deltaeduc ib1.deltafull##ib3.timeperiod i.college##ib3.timeperiod deltau13 delta_kidage if (inwave23==1 & timeperiod==3 & u13delta3==1) | (inwave12==1 & timeperiod==2 & u13delta3==1) || newid:
